# Supplementary material for: Characterization of TelE, a T7SS LXG Effector Exhibiting a Conserved C-Terminal Glycine Zipper Motif Required for Toxicity
Source: Microbiol Spectr. 2023 Jul 11;11(4):e01481-23. doi: 10.1128/spectrum.01481-23 (PMC10434224; doi:10.1128/spectrum.01481-23)

Fig. S1a

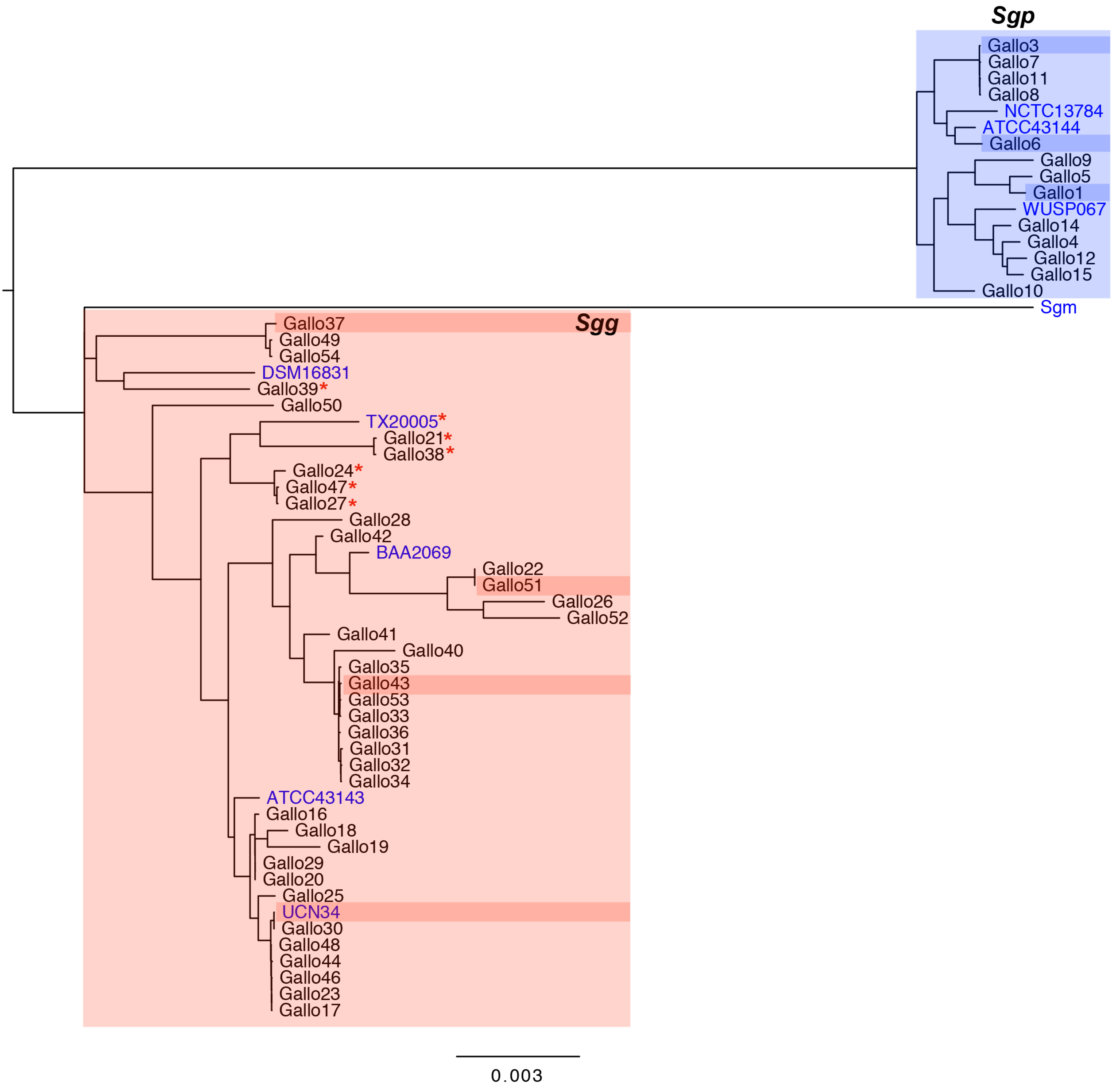

Sgp
Gallo3
Gallo7
Gallo11
Gallo8
NCTC13784
ATCC43144
Gallo6
Gallo9
Gallo5
Gallo1
WUSP067
Gallo14
Gallo4
Gallo12
Gallo15
Gallo10
Sgm
Sgg
Gallo37
Gallo49
Gallo54
DSM16831
Gallo39*
Gallo50
TX20005*
Gallo21*
Gallo38*
Gallo24*
Gallo47*
Gallo27*
Gallo28
Gallo42
BAA2069
Gallo22
Gallo51
Gallo26
Gallo52
Gallo41
Gallo40
Gallo35
Gallo43
Gallo53
Gallo33
Gallo36
Gallo31
Gallo32
Gallo34
ATCC43143
Gallo16
Gallo18
Gallo19
Gallo29
Gallo20
Gallo25
UCN34
Gallo30
Gallo48
Gallo44
Gallo46
Gallo23
Gallo17
0.003

Figure S1b

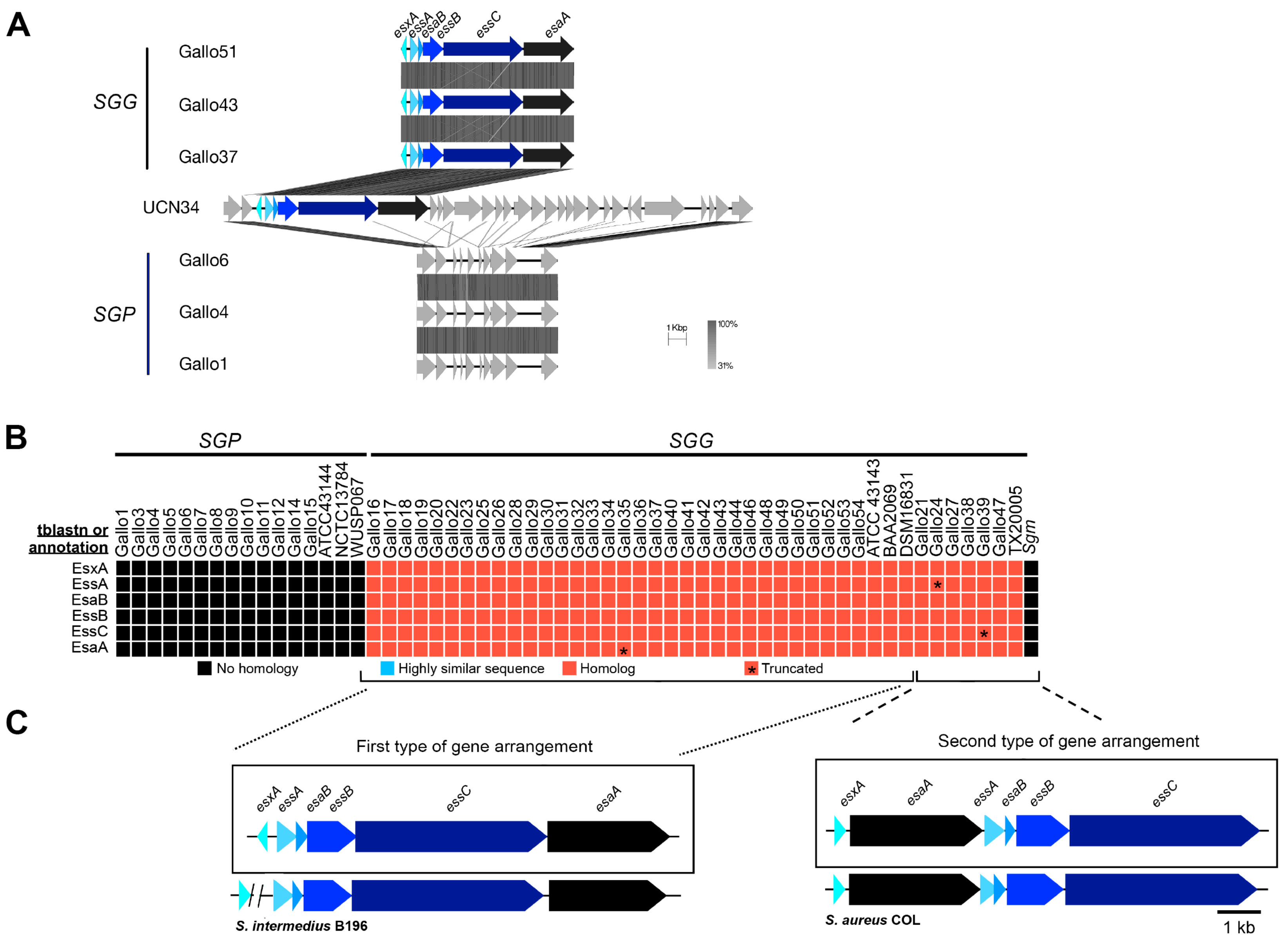
A
SGG
Gallo51
Gallo43
Gallo37
UCN34
SGP
Gallo6
Gallo4
Gallo1
esxA
essA
esaB
essB
essC
esaA
1 Kbp
100%
31%
B
SGP
SGG
tblastn or annotation
EsxA
EssA
EsaB
EssB
EssC
EsaA
Gallo1 Gallo3 Gallo4 Gallo5 Gallo6 Gallo7 Gallo8 Gallo9 Gallo10 Gallo11 Gallo12 Gallo14 Gallo15 ATCC43144 NCTC13784 WUSP067
Gallo16 Gallo17 Gallo18 Gallo19 Gallo20 Gallo22 Gallo23 Gallo25 Gallo26 Gallo28 Gallo29 Gallo30 Gallo31 Gallo32 Gallo33 Gallo34 Gallo35 Gallo36 Gallo37 Gallo40 Gallo41 Gallo42 Gallo43 Gallo44 Gallo46 Gallo48 Gallo49 Gallo50 Gallo51 Gallo52 Gallo53 Gallo54 ATCC 43143 BAA2069 DSM16831 Gallo21 Gallo24 Gallo27 Gallo38 Gallo39 Gallo47 TX20005 Sgm
No homology
Highly similar sequence
Homolog
Truncated
C
First type of gene arrangement
esxA
essA
esaB
essB
essC
esaA
S. intermedius B196
Second type of gene arrangement
esxA
esaA
essA
esaB
essB
essC
S. aureus COL
1 kb

Fig. S2

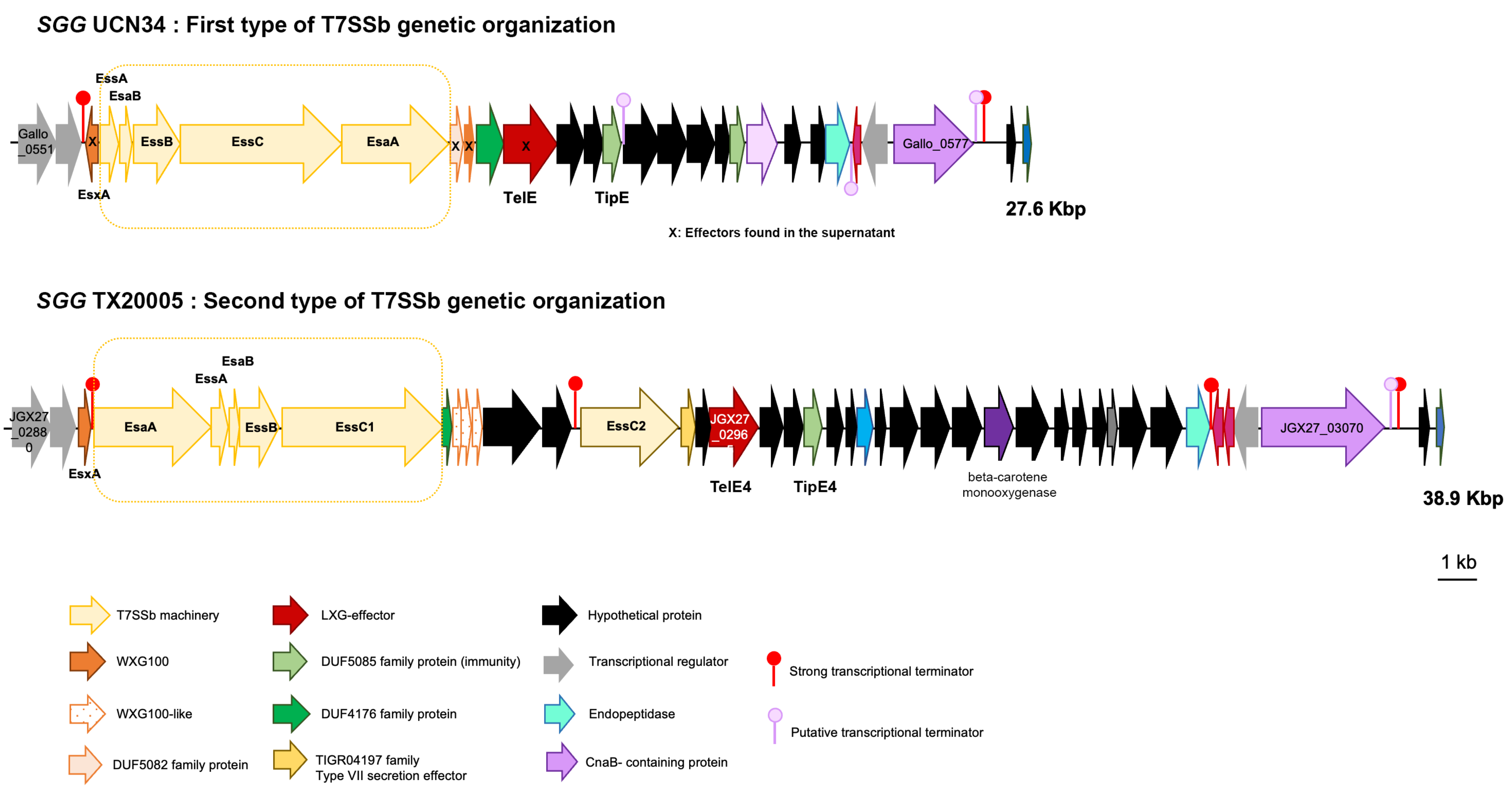

SGG UCN34 : First type of T7SSb genetic organization
Gallo_0551
EssA
EsaB
EsxA
EssB
EssC
EsaA
X
TelE
TipE
Gallo_0577
27.6 Kbp
X: Effectors found in the supernatant
SGG TX20005 : Second type of T7SSb genetic organization
JGX27_02880
EsxA
EsaA
EssA
EsaB
EssB
EssC1
EssC2
JGX27_0296
TelE4
TipE4
beta-carotene monooxygenase
JGX27_03070
38.9 Kbp
1 kb
T7SSb machinery
WXG100
WXG100-like
DUF5082 family protein
LXG-effector
DUF5085 family protein (immunity)
DUF4176 family protein
TIGR04197 family Type VII secretion effector
Hypothetical protein
Transcriptional regulator
Endopeptidase
CnaB- containing protein
Strong transcriptional terminator
Putative transcriptional terminator

Figure S3

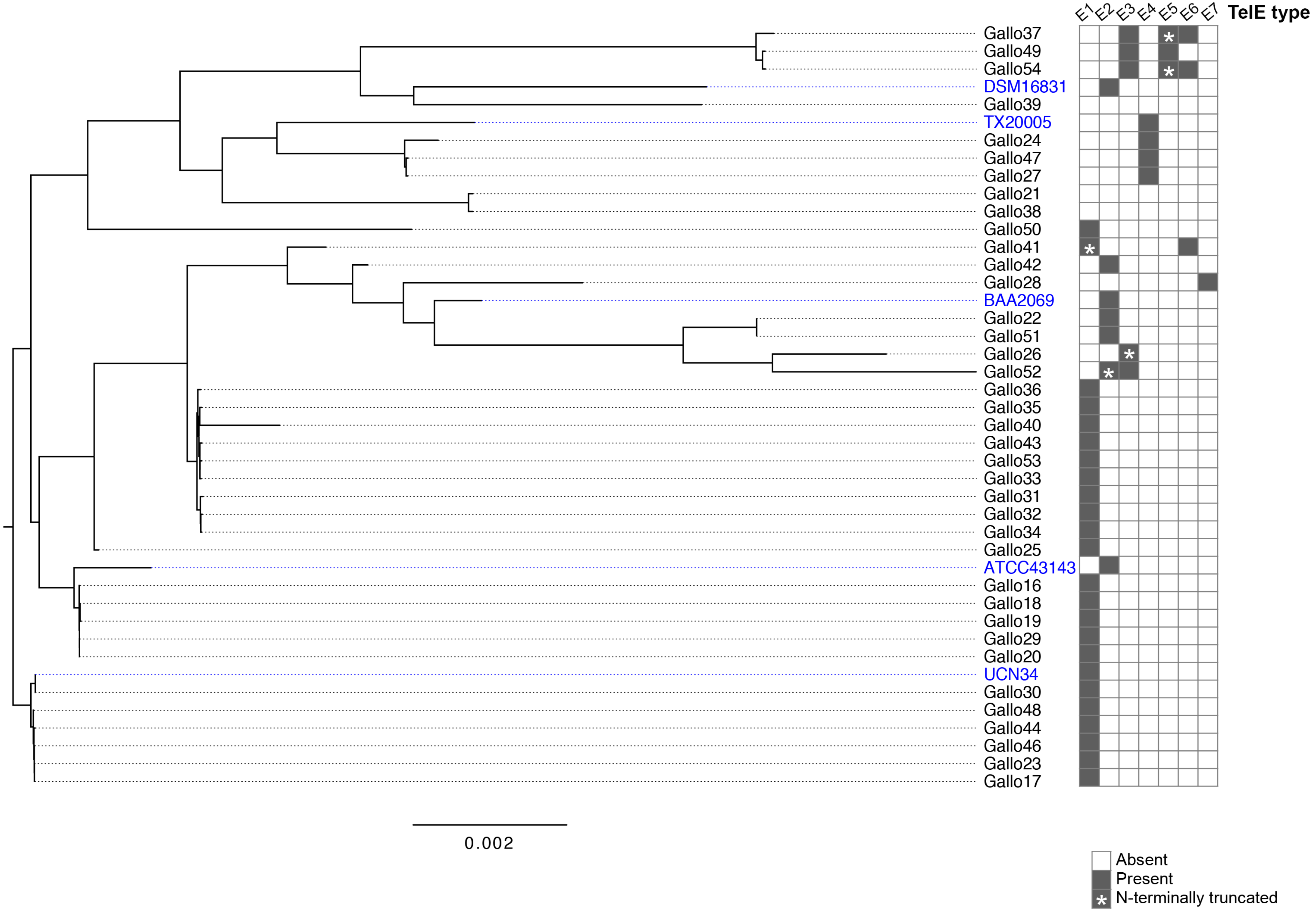
E1
E2
E3
E4
E5
E6
E7
TelE type
Gallo37
Gallo49
Gallo54
DSM16831
Gallo39
TX20005
Gallo24
Gallo47
Gallo27
Gallo21
Gallo38
Gallo50
Gallo41
Gallo42
Gallo28
BAA2069
Gallo22
Gallo51
Gallo26
Gallo52
Gallo36
Gallo35
Gallo40
Gallo43
Gallo53
Gallo33
Gallo31
Gallo32
Gallo34
Gallo25
ATCC43143
Gallo16
Gallo18
Gallo19
Gallo29
Gallo20
UCN34
Gallo30
Gallo48
Gallo44
Gallo46
Gallo23
Gallo17
0.002
Absent
Present
N-terminally truncated

Figure S4

```
                  cov     pid   1 [         .         .         .         .         :         .         .         .         .         1 100
1 TelE1 100.0% 100.0%     M-KIKMSEVLSQKESLASSISSAKSQLESASRKLKSTADSDALQGNVKEAIDNKITNYQVPLLTNYVNSLAVISQGYDNLISTFKETVSETSDSAIIDTD
2 TelE2 100.0%  95.5%     M-KIKMSEVLSQKESLASSISSAKSQLESASRKLKSTADSDALQGNVKEAIDNKITNYQVPLLTNYVNSLAVISQGYDNLISTFKETVSETSDSAIIDTD
3 TelE3  99.6%  95.1%     M-KIKMSEVLSQKESLASSISSAKSQLESASRKLKSTADSDALQGNVKEAIDNKITNYQVPLLTNYVNSLAVISQGYDNLISTFKETVSETSDSAIIDTD
4 TelE4  88.3%  28.6%     MVKMSLGNSTSQGSSTSSISSSRISAYNGAISSLNSFIGASNLQGDAYSSAKSYASTVLIPLIQGAI----LLSEAVSNAVTTFPNRYTSEVAGESLDSE
5 TelE5  99.4%  95.3%     M-KIKMSEVLSQKESLASSISSAKSQLESASRKLKSTADSDALQGNVKEAIDNKITNYQVPLLTNYVNSLAVISQGYDNLISTFKETVSETSDSAIIDTD
6 TelE6  95.3%  57.8%     M-KIKMSEVLSQKESLASSISSAKSQLESASRKLKSTADSDALQGNVKEAIDNKITNYQVPLLTNYVNSLAVISQGYDNLISTFKETVSETSDSAIIDTD
7 TelE7  95.7%  52.1%     M-KIKMSEVLSQKESLASSISSAKSQLESASRKLKSTADSDALQGNVKEAIDNKITNYQVPLLTNYVNSLAVISQGYDNLISTFKETVSETSDSAIIDTD

                  cov     pid 101           .         .         .         .         :         .         .         .         .         2 200
1 TelE1 100.0% 100.0%     VLQQMVDKFDSPLEQLKT-CSDEINKAIDEVADIV--------SLTKVT----TADAISEFKSAKKVLTNTIKDMGIFNSTVFTSEAGDILEEQNTQLSS
2 TelE2 100.0%  95.5%     VLQQMVDKFDSPLEQLKT-CSDEINKAIDEVADIV--------SLTKVT----TADAISEFKSAKKVLTNTIKDMGIFNNTVFTSEAGDILEEQNTQLSS
3 TelE3  99.6%  95.1%     ILQQMVDKFDSPLEQLKT-CSDEINKAIDEVADIV--------SLTKVT----TADAISEFKSAKKVLTNTIKDMGVFNSTVFTSEAGDILEEQNTQLSS
4 TelE4  88.3%  28.6%     VLEAQIATYQAAYDRSSAWLTSEMSKKVLNESSILRAQQSMARNLGKVTELQEKLDKLMAFNASSPSLFDEISGL--------YSAVSQGLSQVNNSFSS
5 TelE5  99.4%  95.3%     VLQQMVDKFDSPLEQLKT-CSDEINKAIDEVADIV--------SLTKVT----TADAISEFKSAKKVLTNTIKDMGIFNSTVFTSEAGDILEEQNTQLSS
6 TelE6  95.3%  57.8%     VLQQMVDKFDSPLEQLKT-CSDEINKAIDEVADIV--------SLTKVT----TADAISEFKSAKKVLTNTIKDMGIFNSTVFTSEAGDILEEQNTQLSS
7 TelE7  95.7%  52.1%     ILQQMVDKFDSPLEQLKT-CSNEINKAIDEVADIV--------SLTKVT----TADAISEFKSAKKVLTNTIKDMGIFNNTVFTSEAGDILEEQNTQLSS

                  cov     pid 201           .         .         .         .         :         .         .         .         .         3 300
1 TelE1 100.0% 100.0%     LSDLGSSSYTSKKAKEFYKDSTFKAEVKEVKSVVNGKARRDQLKNALAKNLYDSKYSGYMLETSGLEVKAGILITS------------------------
2 TelE2 100.0%  95.5%     LSDLGSSSYTSKKAKDFYKDSTFKAEVKEVKSVVNGKARRDQLKNALAKNLYDSKYSGYMLETSGLEVKAGILITS------------------------
3 TelE3  99.6%  95.1%     LSDLGSSSYTSKKAKDFYKDSTFKAEVKEVKSVVNGKARRDQLKNALAKNLYDSKYSGYMLETSGLEVKAGILITS------------------------
4 TelE4  88.3%  28.6%     YN--GTFSLPSQEELAWTTTITTAWEEREANS---------------ASEEFDETIAN-IAEESGLTVEEVMAYVLY-----------------------
5 TelE5  99.4%  95.3%     LSDLGSSSYTSKKAKEFYKDSTFKAEVKEVKSVVNGKARRDQLKNALAKNLYDSKYSGYMLETSGLEVKAGILITS------------------------
6 TelE6  95.3%  57.8%     LSDLGSSSYTGKKAKDFYKDSTFKAEVKEVKSVVNGKARRDQLKNALAKNLYDSKYSGYMMETS---LPTPIMLTL------------------------
7 TelE7  95.7%  52.1%     LSDLGSSSYTSKKAKDFYKDSTFKAEVKEVKSVVNGKARRDQLKNALAKNLYDSKYSGYMMEQTRLEVTVASVTTPYLVGRKYYKKAKKWATSAYAEHRF

                  cov     pid 301           .         .         .         .         :         .         .         .         .         4 400
1 TelE1 100.0% 100.0%     ---------TKKSIGKGKKVVKILDA-TDKALKQK-K------FIGGRELM------------VDSKGRVKVGERF------LYKKPSKGKTKT----VH
2 TelE2 100.0%  95.5%     ---------TKKSIGKGKKVVKILDA-TDKALKQK-K------FIGGRELT------------VDSKGRVKVGERF------LYKKPSKGKTKT----VH
3 TelE3  99.6%  95.1%     ---------TKKSIGKGKKVVKILDA-TDKALKQK-K------FIGGRELT------------VDSKGRVKVGERF------LYKKPSKGKTKT----VH
4 TelE4  88.3%  28.6%     ---------GKKPISYTKKAQKILAT-AIKYQKGK-A------FLKGKEIT------------VEK----------------VYAGTSRESTRVRWNGKP
5 TelE5  99.4%  95.3%     ---------TKKSIGKGKKVVKILDA-TDKALKQK-K------FIGGRELT------------VDSKGRVKVGERF------LYKKPSKGKTKT----VH
6 TelE6  95.3%  57.8%     ---------REKGSDYYKEFQKVMDNDTAKYLSEAFK------FLPSKLIK-----------------SLKQSEGFKEIIWGLAQSGEKGEKAA----TY
7 TelE7  95.7%  52.1%     SKDWMGNTIIKKSEKTVDHALDFIFG-TSKTLKKK-KSHSIVQYHGNKTVTKSFEKLMKTLGETDYSKEIRKSLKT------IEKNGLKGTSKA----KE

                  cov     pid 401           .         .         .         .         :         .         .         .         .         5 500
1 TelE1 100.0% 100.0%     LYS---KGTKEYKKTVGEDF--SFKKTTLYNGRKLKANGKVGID-----WKGVGTSGKEAFKSGLKDNFKSTINPLNEFKGFKEASNFSKAGKVLGFAGT
2 TelE2 100.0%  95.5%     LYS---KGTKEYKKTVGEDF--SFKKTTLYNGRKLKANGKVGID-----WKGVGTSGKEAFKSGLKDNFKSTINPLNEFKGFKEASNFSKAGKVLGFAGT
3 TelE3  99.6%  95.1%     LYS---KGTKEYKKTVGEDF--SFKKTTLYNGRKLKANGKVGID-----WKGVGTSGKEAFKSGLKDNFKSTINPLNEFKGFKEASNFSKAGKVLGFAGT
4 TelE4  88.3%  28.6%     LYD---QETDQLFKS----------------GKDLKEASEIDISKTRYAWTAEGNINFSEMGKAGWSSFKSAIDPLSDFKGWKDASNVTKAGKVLGIAGT
5 TelE5  99.4%  95.3%     LYS---KGTKEYKKTVGEDF--SFKKTTLYNGRKLKANGKVGID-----WKGVGTSGKEAFKSGLKDNFKSTINPLNE---FKEASNFSKAGKVLGFAGT
6 TelE6  95.3%  57.8%     VL----KGLMKY-----EEFGNAIKNTKL--GTLIAGTSKVFDG-----AKKVADPIKSFVKEGLKSTKFYKAISETATAGGEVSGTLNFLGKGLKFAGK
7 TelE7  95.7%  52.1%     IAKGVLKGTKSNLISEAEE---AFNVKTVKN--VAKATKKTGQI-----TKGVKTAFKES----------------NVVTALKTGSKFTKGVAVLNVIDG

                  cov     pid 501           .         .         .         .         :         .         .         .         .         6 600
1 TelE1 100.0% 100.0%     VMTIGSNIKTDFID-DRRSS------TKEKVRNFAVD------------TTVDVVSSSSAAAAGAAVGTLVGGPVGTVVGAAVGIGFS------------
2 TelE2 100.0%  95.5%     VMTIGSNIKTDFID-DRRSS------TKEKVRNFAVD------------TTVDVVSSSSAAATGAAIGTLVGGPLGTVAGAIVGIAVS------------
3 TelE3  99.6%  95.1%     VMTIGSNIKTDFID-DRKSS------TKEKVRNFAVD------------TTVDVVSSSSAAAAGAAVGTLVGGPLGTVVGAAVGIAIS------------
4 TelE4  88.3%  28.6%     AMTVINNVNADFINADGGIN------SAQNWGNFAVD------------TGVDLASGAGAAAIGAAAGSLVAPPLGTVVGAGVGMLVS------------
5 TelE5  99.4%  95.3%     VMTIGSNIKTDFID-DRRSS------TKEKVRNFAVD------------TTVDVVSSSSAAAAGAAVGTLVGGPLGTVVGAAAGIAVS------------
6 TelE6  95.3%  57.8%     AATV-----VTFAD-VAISG------VSGGVKEFAKTGDVGKGVIAGTFSAVKSVGPLEGATIGAAVG---GGFPGAVIGAGVGLGIYILDKTGVLD---
7 TelE7  95.7%  52.1%     VAETFDNVSKNKTQ-AKKQGLQGAEVSASVATGFAVDA---------AKATVKGVASTAAATAGAALA---EAGIGLLAGAGFTVGAPVL-ITGVAAIAI
                                                                                     ____________________________
                                                                                         Glycine rich motif

                  cov     pid 601           .         .         .         .         :    ] 654
1 TelE1 100.0% 100.0%     --------VAMNHRVKFLGG---------KSLTEVTK---DSLKGVAG-KFGL-
2 TelE2 100.0%  95.5%     --------EFMNTNLEILGN---------KSLTGVAK---SGLKGLAG-KFGL-
3 TelE3  99.6%  95.1%     --------KAFDHKW--GGK---------NSINDYAK---NSLKGLAG-KFGL-
4 TelE4  88.3%  28.6%     --------WGMNQDW--GGG---------KSVTTWAK---DSLKGLFN------
5 TelE5  99.4%  95.3%     --------WALNKGFFGKGK---------KSINDCAK---DGLKGLAG-KFGL-
6 TelE6  95.3%  57.8%     --------TAEDLTMKFYDKSKEVVGKAVKSVGEAIS---TTFGGIGK-ALGFG
7 TelE7  95.7%  52.1%     GALAAKGVEIADKKFNITGNIK-------STANSFIKGCGDFIKGMKGWNHGVN
```

Figure S5

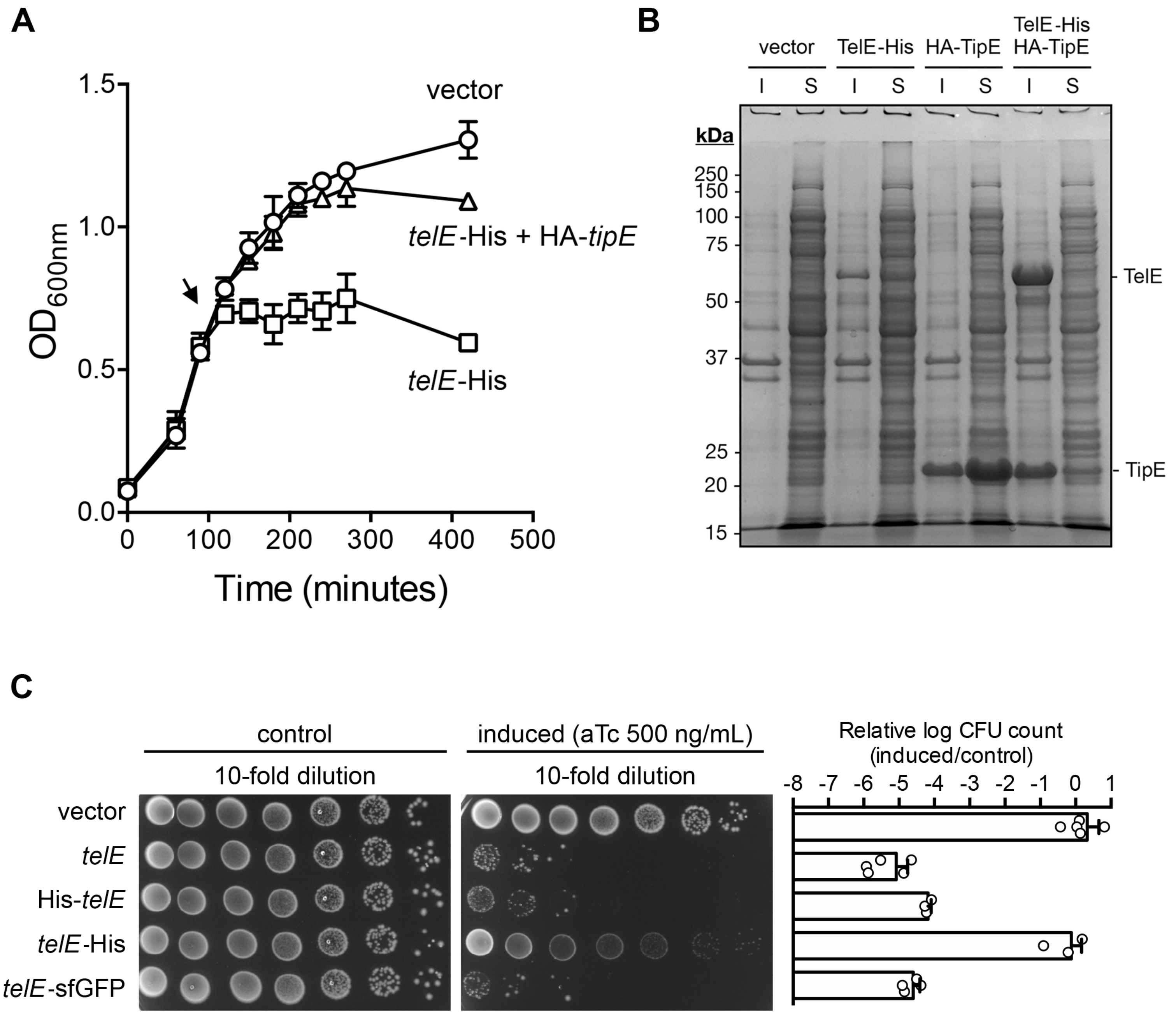
A
vector
telE-His + HA-tipE
telE-His
OD600nm
Time (minutes)
0.0
0.5
1.0
1.5
0
100
200
300
400
500
B
vector
TelE-His
HA-TipE
TelE-His
HA-TipE
I S I S I S I S
kDa
250
150
100
75
50
37
25
20
15
- TelE
- TipE
C
control
10-fold dilution
induced (aTc 500 ng/mL)
10-fold dilution
vector
telE
His-telE
telE-His
telE-sfGFP
Relative log CFU count
(induced/control)
-8 -7 -6 -5 -4 -3 -2 -1 0 1

Fig. S6

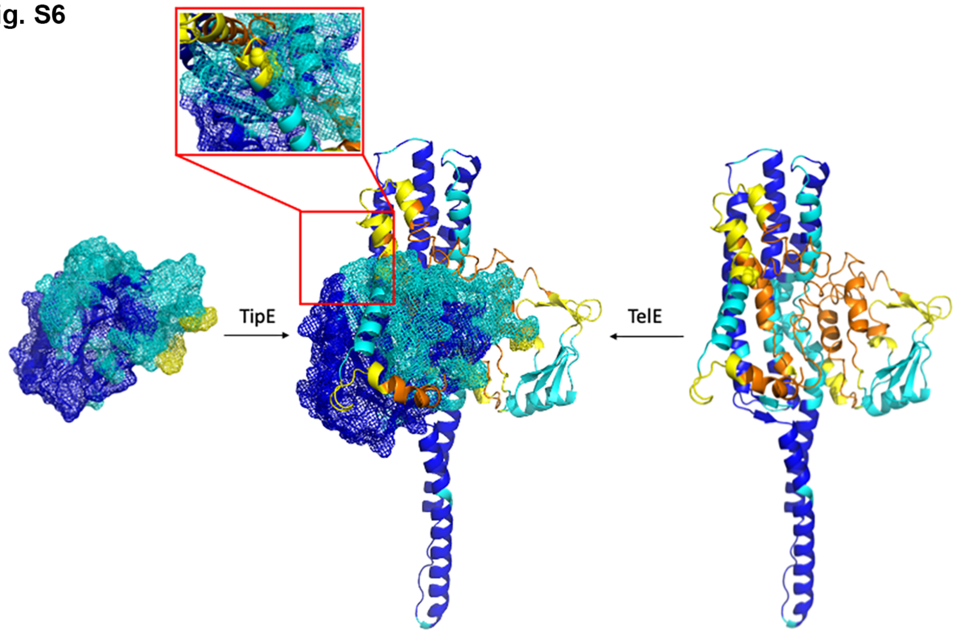

Supplement: Supplemental file 1 — Fig. S1 to Fig. S6. Download spectrum.01481-23-s0001.pdf, PDF file, 4.6 MB [file spectrum.01481-23-s0001.pdf]
