# Supplementary material for: Characterization of TelE, a T7SS LXG Effector Exhibiting a Conserved C-Terminal Glycine Zipper Motif Required for Toxicity
Source: Microbiol Spectr. 2023 Jul 11;11(4):e01481-23. doi: 10.1128/spectrum.01481-23 (PMC10434224; doi:10.1128/spectrum.01481-23)
Supplement: Supplemental file 2 — Supplemental legends. Download spectrum.01481-23-s0002.docx, DOCX file, 0.02 MB [file spectrum.01481-23-s0002.docx]

**SUPPLEMENTARY FIGURES LEGEND**

**Fig. S1a. Phylogenetic tree of the clinical isolates sequenced in this study.** Phylogenetic inference of the isolates was performed using approximate-maximum-likelihood algorithm based on the core-genome alignment using Parsnp version 1.2. Scale bar represents substitution per variant site within the core genome. The isolates sequenced in this study were labeled in black font, whereas the reference strains *SGP* (NCTC13784, ATCC43144 and WUSP067) and *SGG* (DSM16831, ATCC43143, BAA2069 and UCN34) with complete genomes available on NCBI GenBank, and a draft genome of *SGM* (strain ACA-DC-206) were labeled in blue font. Genomes used for comparative genomics analysis shown in Fig. 1A were highlighted with darker color shades. Bootstrap value for all the major branches ranges from 0.97 to 1. Genomes labelled with asterisk contain a different T7SSb gene locus arrangement than the UCN34 reference strain.

**Fig. S1b.** **Comparison of the T7SSb locus in several *SGG* and *SGP* clinical isolates**. (A) A pairwise nucleotide sequence comparison of the genomic region of interest. Each of the T7SSb genes (arranged in the order of *esxA, essA, esaB, essB, essC, esaA*) was represented by colored arrows. Light gray arrows represent other open reading frames in this genomic region. Gray shading shows the similarities between the sequences. (B) Summary of BLAST search using NCBI tBLASTn program, queried against the protein sequences of each T7SSb components (EsxA, EssA, EsaB, EssB, EssC, EsaA) from the reference genome UCN34. The identification of homologs was based on RAST annotation, or significant hits from the BLAST search. (C) The general gene arrangement of the T7SSb gene locus in *SGG*, *S. intermedius* and *S. aureus*. Each T7SSb genes was color-coded and labelled accordingly. The open reading frames flanked by *esxA* and *essA* in the *S. intermedius* B196 were excluded in this figure for a clear presentation of the T7SSb gene locus.

**Figure S2**. **Detailed comparison of the whole T7SSb locus in two representative strains UCN34 and TX20005.**

**Figure S3. The prevalence of TelE homologs among *SGG* isolates.** The phylogenetic inference of the *SGG* isolates was shown on the left. Genomes in black font represent the clinical isolates sequenced in this study, whereas the genomes in blue font represent the complete genomes publicly available. Scale bar represents substitution per variant site within the core genome. Table on the right summarized the distribution of TelE homologs among *SGG* isolates. Based on the C-terminal sequence (shown in Figure 3C), the identified TelE homologs were broadly categorized into 7 TelE variants and named as TelE1 to TelE7, where TelE1 refers to the reference TelE from strain UCN34. Bootstrap values of all the major branches are 1.

**Figure S4. Multiple sequence alignment of various TelE subtypes.** Amino acid sequences of TelE1 to TelE7 subtypes were aligned using online MAFFT server version 7 with E-INS-i iterative refinement method that assumes all the sequences shared the same conserved motifs. The aligned sequences were viewed using MView (75) available on EMBL-EBI.

**Figure S5. TipE counteracts TelE toxicity.** (A) Viability of *E. coli* cells harboring an empty vector (pTCVerm-P_tetO_) or a vector encoding *telE* fused with epitope tags or superfolder GFP with the expression inducible by anhydrotetracycline (aTc). Bar chart on the right showed the logarithm-transformed CFU count of the cells expressing the *telE* variants in relative to the control. Error bars represent mean + standard deviation (n ≥ 3). (B) Growth kinetics of the *E. coli* BL21(DE3) harboring either the empty vector or a vector for IPTG-inducible expression of *telE*-His or co-expression of *telE*-His and HA-*tipE.* Arrow indicates the time point when the gene expression was induced. (C) Total protein collected from the respective lysates was resolved by SDS-PAGE and stained with Coomassie blue. I, insoluble lysate; S, soluble lysate. Arrow indicates the protein band corresponding to TelE and TipE, respectively.

**Fig. S6 An Alpha Fold Model of the TelE-TipE interaction.** A representative AlphaFold2 prediction of TelE-TipE complex showing the pLDDT confidence score (scale from 0 to 100). Based on this metric, the model is colored in blue where the per-residue confidence is high (pLDDT > 90), in cyan when good (pLDDT > 70), in yellow when low (pLDDT > 50), and in orange when very low (pLDDT < 50) (76). To better distinguish the two subunits in the complex, TipE (left side) is in the 'mesh' PyMOL representation, while TelE is depicted in the 'cartoon' form (right side). An inset on the top highlights the glycine 470 of TelE that is surrounded by TipE in the model.
