# Supplementary material for: Characterization of TelE, a T7SS LXG Effector Exhibiting a Conserved C-Terminal Glycine Zipper Motif Required for Toxicity
Source: Microbiol Spectr. 2023 Jul 11;11(4):e01481-23. doi: 10.1128/spectrum.01481-23 (PMC10434224; doi:10.1128/spectrum.01481-23)
Supplement: Supplemental file 3 — Table S1. Download spectrum.01481-23-s0003.docx, DOCX file, 0.03 MB [file spectrum.01481-23-s0003.docx]

**Table S1. Strains, plasmids and primers used in this study.**

| **Strains** |  |  |
| --- | --- | --- |
|  | Description | Reference |
| *Sgg* UCN34 | A clinical strain isolated from an infective endocarditis patient who later diagnosed with CRC | (1) |
| *S. agalactiae* NEM316 | MLST-23, serotype III isolated from neonate blood culture | (2) |
| *E. coli* DH5𝝰 | *deoR endA1 gyrA96 hsdR17 ∆*(*lac*)*U169 recA1 relA1 supE44 thi-1* (ϕ80 *lacZ∆*M15) | Lab collection |
| *E. coli* BL21(DE3) | *gal hsdS*_B_ *ompT* | Lab collection |
| *E. coli* GM48 | *thr-1* *araC14 leuB6 fhuA31 lacY1 tsx-78 glnX44 galK2 galT22 LAM- dcm-6 dam-3 thiE1* | Lab collection |
| **Plasmids** |  |  |
|  | Description | Reference |
| pG1 | Temperature sensitive vector to clone in PCR fragments at *Sma*I site; Erm^R^ | (3) |
| pG1-*essC*KO | to generate a deletion mutant of *essC* (*gallo_0557*) in *Sgg* UCN34 | This study |
| pTCV*erm*-*ptetO* | *E.* coli/*Streptococcus*/*Enterococcus* shuttle vector for inducible gene expression using anhydrotetracycline; Kan^R^ Erm^R^ | (3) |
| pTCV*erm*-*ptetO*-*esxA* | pTCV*erm*-*ptetO* containing *esxA* ORF (*Gallo_0553*) at *Bam*HI / *Sph*I sites | This study |
| pTCV*erm*-*ptetO*-*gallo0559* | pTCV*erm*-*ptetO* containing *Gallo_0559* ORF at *Bam*HI / *Sph*I sites | This study |
| pTCV*erm*-*ptetO*-*gallo0560* | pTCV*erm*-*ptetO* containing *Gallo_0560* ORF at *Bam*HI / *Sph*I sites | This study |
| pTCV*erm*-*ptetO*-*telC1* | pTCV*erm*-*ptetO* containing *telC1* ORF (*Gallo_1068*) at *Bam*HI / *Pst*I sites | This study |
| pTCV*erm*-*ptetO*-*telC2* | pTCV*erm*-*ptetO* containing *telC2* ORF (*Gallo_1574*) at *Bam*HI/ *Pst*I sites | This study |
| pTCV*erm*-*ptetO*-*telE* | pTCV*erm*-*ptetO* containing *telE* ORF (*Gallo_0562*) at *Bam*HI / *Sph*I sites | This study |
| pTCV*erm*-*ptetO*-*telE2* | pTCV*erm*-*ptetO* containing *telE2* ORF (amplified from Gallo42 isolate) at *Bam*HI/ *Sph*I sites | This study |
| pTCV*erm*-*ptetO*-*telE3* | pTCV*erm*-*ptetO* containing *telE3* ORF (amplified from Gallo37 isolate) at *Bam*HI /*Sph*I sites | This study |
| pTCV*erm*-*ptetO*-*telE4* | pTCV*erm*-*ptetO* containing *telE4* ORF (amplified from Gallo47 isolate) at *Bam*HI/ *Sph*I sites | This study |
| pTCV*erm*-*ptetO*-*telE5* | pTCV*erm*-*ptetO* containing *telE5* ORF (amplified from Gallo49 isolate) at *Bam*HI / *Sph*I sites | This study |
| pTCV*erm*-*ptetO*-*telE6* | pTCV*erm*-*ptetO* containing *telE6* ORF (amplified from Gallo54 isolate) at *Bam*HI/ *Sph*I sites | This study |
| pTCV*erm*-*ptetO*-*telE7* | pTCV*erm*-*ptetO* containing *telE7* ORF (amplified from Gallo28 isolate) at *Bam*HI / *Sph*I sites | This study |
| pTCV*erm*-*ptetO*-*telE-G458V* | pTCV*erm*-*ptetO* containing *telE* ORF (*Gallo_0562*) with base substitution of G to T at nucleotide position 1373 at *Bam*HI /*Sph*I sites | This study |
| pTCV*erm*-*ptetO*-*telE-G466V* | pTCV*erm*-*ptetO* containing *telE* ORF (*Gallo_0562*) with base substitution of G to T at nucleotide position 1397 at *Bam*HI / *Sph*I sites | This study |
| pTCV*erm*-*ptetO*-*telE-G470V* | pTCV*erm*-*ptetO* containing *telE* ORF (*Gallo_0562*) with base substitution of G to T at nucleotide position 1409 at *Bam*HI /*Sph*I sites | This study |
| pTCV*erm*-*ptetO*-*telE-G474V* | pTCV*erm*-*ptetO* containing *telE* ORF (*Gallo_0562*) with base substitution of G to T at nucleotide position 1421 at *Bam*HI/ *Sph*I sites | This study |
| pTCV*erm*-*ptetO*-*telE-G478V* | pTCV*erm*-*ptetO* containing *telE* ORF (*Gallo_0562*) with base substitution of G to T at nucleotide position 1433 at *Bam*HI / *Sph*I sites | This study |
| pTCV*erm*-*ptetO*-*telE-G480V* | pTCV*erm*-*ptetO* containing *telE* ORF (*Gallo_0562*) with base substitution of G to T at nucleotide position 1439 at *Bam*HI/*Sph*I sites | This study |
| pTCV*erm*-*ptetO*-*telE-His* | pTCV*erm*-*ptetO* containing *telE* ORF (*Gallo_0562*) with a 3’ hexahistidine tag at *Bam*HI /*Sph*I sites | This study |
| pTCV*erm*-*ptetO*-*His-telE* | pTCV*erm*-*ptetO* containing *telE* ORF (*Gallo_0562*) with a 5’ hexahistidine tag at *Bam*HI / *Sph*I sites | This study |
| pTCV*erm*-*ptetO*-*telE*-*SGGsfGFP* | pTCV*erm*-*ptetO* containing *telE* ORF (*Gallo_0562*) with a 3’ *Streptococcus gallolyticus* codon-optimized superfolder GFP (SGGsfGFP) tag at *Bam*HI / *Sph*I sites | This study |
| pJN105 | *E. coli/Pseudomonas* shuttle vector for inducible gene expression using arabinose; Gm^R^ | (4) |
| pJN105-*Gallo0563* | pJN105 containing *Gallo_0563* ORF at *Nhe*I / *Xba*I site, downstream of pBAD promoter | This study |
| pJN105-*Gallo0564* | pJN105 containing *Gallo_0564* ORF at *Nhe*I / *Xba*I site, downstream of pBAD promoter | This study |
| pJN105-*tipE* | pJN105 containing *Gallo_0565* ORF at *Nhe*I/ *Xba*I site, downstream of pBAD promoter | This study |
| pJN105-*Gallo0570* | pJN105 containing *Gallo_0570* ORF at *Nhe*I/ *Xba*I site, downstream of pBAD promoter | This study |
| pET28b | *E. coli* recombinant protein expression vector; Kan^R^ | Lab collection |
| pET28b-*telE-His* | pET28b containing *telE* ORF without a start codon and a stop codon at *Nco*I / *Xho*I sites | This study |
| pET28b*-HA-tipE-T7-telE-His* | pET28b-*telE-His* containing N-terminally HA-tagged TipE ORF followed by a T7 promoter at *Nco*I site | This study |
| pET28b-*HA-tipE* | pET28b containing *tipE* ORF without start codon at *Nco*I */ Xho*I sites | This study |
| pET28b-*telE_G470V_-His* | pET28b containing *telE_G470V_* ORF without a start codon and a stop codon at *Nco*I / *Xho*I sites | This study |
|  |  |  |
| **Primers** |  |  |
|  | Sequence (5’ to 3’) |  |
| **Knockout mutants** | | |
| essCKO_up | GATCTTACACGAGAGAC //  CACCATAACGTGTACGGTGATAGTAAGTCATATTATC | This study |
| essCKO_down | GATAATATGACTTACTATCACCGTACACGTTATGGTG //  GCTCACTTCAGCTTGG | This study |
|  |  |  |
| **Gene expression in pTCV*erm*-*ptetO*** | | |
| BamHI/SphI_Gallo0553 | TATA**GGATCC**AGGAGATTTTTATGACC //  TATA**GCATGC**TTAGTTAAGTCCAAATGAAG | This study |
| *Bam*HI/*Sph*I_Gallo0559 | TATA**GGATCC**ATGGGAGAATGTTGTGG //  TATA**GCATGC**TTACATACTACCTGCTGC | This study |
| *Bam*HI/*Sph*I_Gallo0560 | TATA**GGATCC**TGTAAAGGGAGATTTTATGG //  TATA**GCATGC**TTATTTGCCTCCTTTACTAC | This study |
| *Bam*HI/*Pst*I_TelC1 | TATA**GGATCC**CCTCTTGCGAAAGGAG //  TATA**CTGCAG**TCATTAGTTGTCCTCTCC | This study |
| *Bam*HI/*Pst*I_TelC2 | TATA**GGATCC**CCATTTACGTTGCTAAGC //  TATA**CTGCAG**CTATTCTCCTACGGCTTC | This study |
| *Bam*HI/*Sph*I_TelE | TATA**GGATCC**GTGATGTATCCAAAGG //  TATA**GCATGC**TCATAATCCAAATTTACC | This study |
| *Bam*HI/*Sph*I_TelE2 | TATA**GGATCC**GTGATGTATCCAAAGGGAG //  TATA**GCATGC**TCATAATCCAAATTTCCCTGC | This study |
| *Bam*HI/*Sph*I_TelE3 | TATA**GGATCC**AACGATTACATTGGGAGAG //  TATA**GCATGC**TCATAATCCAAATTTCCCTGC | This study |
| *Bam*HI/*Sph*I_TelE4 | TATA**GGATCC**GAAGAGGAACGAAAGG //  TATA**GCATGC**TTAATTAAATAATCCTTTCAAAC | This study |
| *Bam*HI/*Sph*I_TelE5 | TATA**GGATCC**AACGATTACATTGGGAGAG //  TATA**GCATGC**TCATAACCCAAACTTACCTGC | This study |
| *Bam*HI/*Sph*I_TelE6 | TATA**GGATCC**AACGATTACATTGGGAGAG //  TATA**GCATGC**TCACCCAAAACCTAGCG | This study |
| *Bam*HI/*Sph*I_TelE7 | TATA**GGATCC**AACGATTACATTGGGAGAG //  TATA**GCATGC**TCAATTAACCCCATGGTTC | This study |
| *Bam*HI_TelE-G458V_up | TATA**GGATCC**GTGATGTATCCAAAGG //  CTGCTGCTACTGCAG | This study |
| *Sph*I_TelE-G458V_down | CTGCAGTAGCAGCAG //  TATA**GCATGC**TCATAATCCAAATTTACC | This study |
| *Bam*HI_TelE-G466V_up | TATA**GGATCC**GTGATGTATCCAAAGG //  GGTCCAACTACTAAAG | This study |
| *Sph*I_TelE-G466V_down | CTTTAGTAGTTGGACC //  TATA**GCATGC**TCATAATCCAAATTTACC | This study |
| *Bam*HI_TelE-G470V_up | TATA**GGATCC**GTGATGTATCCAAAGG //  CACAGTTACAACTGG | This study |
| *Sph*I_TelE-G470V_down | CCAGTTGTAACTGTG //  TATA**GCATGC**TCATAATCCAAATTTACC | This study |
| *Bam*HI_TelE-G474V_up | TATA**GGATCC**GTGATGTATCCAAAGG //  GCCACTACCACAGTT | This study |
| *Sph*I_TelE-G474V_down | AACTGTGGTAGTGGC //  TATA**GCATGC**TCATAATCCAAATTTACC | This study |
| *Bam*HI_TelE-G478V_up | TATA**GGATCC**GTGATGTATCCAAAGG //  CCAATAACGACTGCG | This study |
| *Sph*I_TelE-G478V_down | CGCAGTCGTTATTGG //  TATA**GCATGC**TCATAATCCAAATTTACC | This study |
| *Bam*HI_TelE-G480V_up | TATA**GGATCC**GTGATGTATCCAAAGG //  CTACAGAAAAGACAATAC | This study |
| *Sph*I_TelE-G480V_down | GTATTGTCTTTTCTGTAG //  TATA**GCATGC**TCATAATCCAAATTTACC | This study |
| *Bam*HI_TelE-GFP_up | TATA**GGATCC**GTGATGTATCCAAAGG //  CCTTTTGATAATCCAAATTTACCC | This study |
| *Sph*I_TelE-GFP_down | GGATTATCAAAAGGTGAAGAATTG //  TATA**GCATGC**TTATTTATACAATTCATCCATAC | This study |
| *Bam*HI/*Sph*I_TelE-His | TATA**GGATCC**GTGATGTATCCAAAGG //  TATA**GCATGC**TCA*GTGATGATGATGATGATG*TAATCCAAATTTACCC | This study |
| *Bam*HI/*Sph*I_His-TelE | TATA**GGATCC**CCAAAGGGAGAGATTTATG*CATCATCATCATCATCAC*AAGATTAAGATGAGCG //  TATA**GCATGC**TCATAATCCAAATTTACC | This study |
|  |  |  |
| Gene expression in pJN105 | | |
| *Nhe*I/XbaI_Gallo0563 | GCAT**GCTAGC**GATAGTTTGAAAGGTGTAG //  GCAT**TCTAGA**CTAAAGCTCATCAATCTC | This study |
| *Nhe*I/*Xba*I_Gallo0564 | GCAT**GCTAGC**GAAGAGATTGATGAGC //  GCAT**TCTAGA**TTAGTCTACCTCCAA | This study |
| *Nhe*I/*Xba*I_TipE | GCAC**GCTAGC**GTATACAGTGATTTGGAG //  GCAC**TCTAGA**TTAATACGCATACCCAAC | This study |
| *Nhe*I/*Xba*I_Gallo0570 | GCAC**GCTAGC**GCAGATATTATTATTCCAG //  GCAC**TCTAGA**CTACTCAAAACGAACAC | This study |
|  |  |  |
| Gene expression in pET28b | | |
| *Nco*I/*Xho*I_TelE-His | TATA**CCATGG**TGAAGATTAAGATGAGCGAG //  TATA**CTCGAG**TAATCCAAATTTACCCGCTAC | This study |
| *Nco*I/*Xho*I_HA-TipE | GCAT**CCATGG**GC*TACCCATACGATGTTCCAGATTACGCT*GAATATACTGTAGCTGAGG //  TATA**CTCGAG**TTAATACGCATACCCAAC | This study |
| *Nco*I_HA-TipE-T7_up | GCAT**CCATGG**GC*TACCCATACGATGTTCCAGATTACGCT*GAATATACTGTAGCTGAGG //  GGATCGTTAATACGCATACCCAAC | This study |
| *Nco*I_HA-TipE-T7_down | GTATTAACGATCCCGCGAAATTAATAC //  CTGC**CCATGG**TATATCTCC | This study |

Underlined, overlapping region for overlap extension PCR; bold, restrictive enzyme recognition sequence; italicized, epitope tag sequence

**Reference**

1. Rusniok C, Couve E, Da Cunha V, El Gana R, Zidane N, Bouchier C, et al. Genome sequence of Streptococcus gallolyticus: insights into its adaptation to the bovine rumen and its ability to cause endocarditis. J Bacteriol. 2010;192(8):2266-76.

2. Glaser P, Rusniok C, Buchrieser C, Chevalier F, Frangeul L, Msadek T, et al. Genome sequence of Streptococcus agalactiae, a pathogen causing invasive neonatal disease. Mol Microbiol. 2002;45(6):1499-513.

3. Danne C, Guerillot R, Glaser P, Trieu-Cuot P, Dramsi S. Construction of isogenic mutants in Streptococcus gallolyticus based on the development of new mobilizable vectors. Res Microbiol. 2013;164(10):973-8.

4. Newman JR, Fuqua C. Broad-host-range expression vectors that carry the L-arabinose-inducible Escherichia coli araBAD promoter and the araC regulator. Gene. 1999;227(2):197-203.
